# Evolution, current status, and future trends of maxillary skeletal expansion: a bibliometric analysis

**DOI:** 10.1007/s00784-023-05430-3

**Published:** 2023-12-22

**Authors:** Zhiyuan Feng, Minmin Si, Hao Fan, Yin Zhang, Rui Yuan, Zhaonan Hao

**Affiliations:** 1 Department of Orthodontics, Shanxi Provincial People’s Hospital, The Fifth Clinical Medical College of Shanxi Medical University, Taiyuan, China; 2 School and Hospital of Stomatology, Shanxi Medical University, Taiyuan, China; 3 School of Information Engineering, Chang’an University, Xi’an, China

**Keywords:** Maxillary expansion, Skeletal expansion, Bibliometric analysis, Nasal cavity, Upper airway

## Abstract

**Objectives:**

The study aims to conduct a bibliometric analysis on maxillary skeletal expansion to elucidate the evolution and current status and predict future research hotspots and trends.

**Material and methods:**

A search was conducted in the Web of Science Core Collection at the University of Hong Kong’s electronic library using the query “(TS = maxillary expansion) AND (TS = skeletal expansion).” The resulting literature data were imported into CiteSpace 6.2.R4 and VOS viewer software to analyze authorship, countries, institutions, keywords, etc.

**Results:**

A total of 923 articles were analyzed. The research in this field has shown a steady growth, with a significant increase since 2019. The USA and Italy have played prominent roles in contributing to the publication volume and strengthening collaborative exchanges. Clustering labels provide directions for in-depth analysis of the literature.

**Conclusions:**

(1) MARPE (miniscrew-assisted rapid palatal expansion) and SARME (surgically assisted rapid maxillary expansion) have gained widespread attention and become research hotspots due to their applicability in adults whose growth and development have ceased, while still producing favorable skeletal effects.

(2) In addition to widening the maxillary arch, maxillary expansion techniques have shown significant effects on increasing nasal cavity width and volume. However, there is still controversy regarding whether they can effectively improve the deviated nasal septum.

(3) Maxillary skeletal expansion techniques have been shown to increase upper airway volume and improve breathing, making them potentially valuable in the treatment of obstructive sleep apnea (OSA).

**Clinical relevance:**

This study can provide cutting-edge clinical recommendations for healthcare professionals to better formulate clinical strategies.

## Introduction

Maxillary transverse deficiency is a common malocclusion often accompanied by unilateral or bilateral posterior crossbite [1]. Its prevalence ranges from 8 to 22% [2]. Since transverse development occurs earlier than vertical and sagittal development, insufficient transverse growth can also affect craniofacial development [3]. In addition, it can impact aesthetics and other functions such as excessive buccal corridor spaces, dental crowding, and reduced nasal cavity volume leading to breathing difficulties [2, 4]. Therefore, timely correction of maxillary transverse deficiency is crucial for harmonizing the relationship between the upper and lower jaws and improving aesthetics and functionality.

The main effective methods for treating maxillary constriction involve palatal expansion achieved through orthopedic and orthodontic tooth movement [5]. Based on the mode of anchorage, these methods can be classified as tooth-borne, tooth-bone mixed-borne, and bone-borne appliances [2]. Currently, rapid maxillary expansion (RME) has become a widely accepted and established treatment modality for correcting maxillary constriction in patients during the peak period of growth and development. However, traditional tooth-borne RME often presents various side effects such as restricted skeletal movement, dental tipping, root resorption, adverse periodontal effects (e.g., dehiscence), and lack of long-term stability [1]. In contrast, tooth-bone mixed-borne or bone-borne RME has demonstrated significant advantages in reducing dental tipping [6], increasing bone sutures’ opening, and decreasing nasal airway resistance [6]. In recent years, there has been a growing number of clinical studies and reviews related to maxillary skeletal expansion. However, there is a lack of comprehensive quantitative analysis, such as scientometrics or bibliometrics [7]. Scientometrics involves the use of statistical techniques to quantitatively analyze data related to authors, countries, institutions, and more, providing preliminary insights into the evolution and advancements in a specific field. Citation analysis is a commonly used method in bibliometric research to assess the impact of publications [7]. CiteSpace and VOSviewer are popular bibliometric software tools used to visualize literature data and analyze research trends. Compared to traditional descriptive reviews, bibliometric analysis offers significant advantages in rapidly identifying key information and guiding future research directions [8].

Therefore, the purpose of this study is to conduct a bibliometric analysis of the literature on maxillary skeletal expansion. It aims to elucidate the evolution and current status of scientific production in this field, demonstrate the contributions and collaborative relationships among authors, countries, and institutions involved, identify influential journals, and reveal research hotspots and trends through keywords and citation analysis. The findings of this study will provide references for future research in this area.

## Material and methods

### Data collection

A search was conducted in the Web of Science Core Collection at the University of Hong Kong’s electronic library using the query “(TS = maxillary expansion) AND (TS = skeletal expansion),” with a time span from 1993–01-01 to 2023–06-30. The document types included articles and reviews, while other criteria such as language were not restricted.

### Data screening

**Fig. 1:**
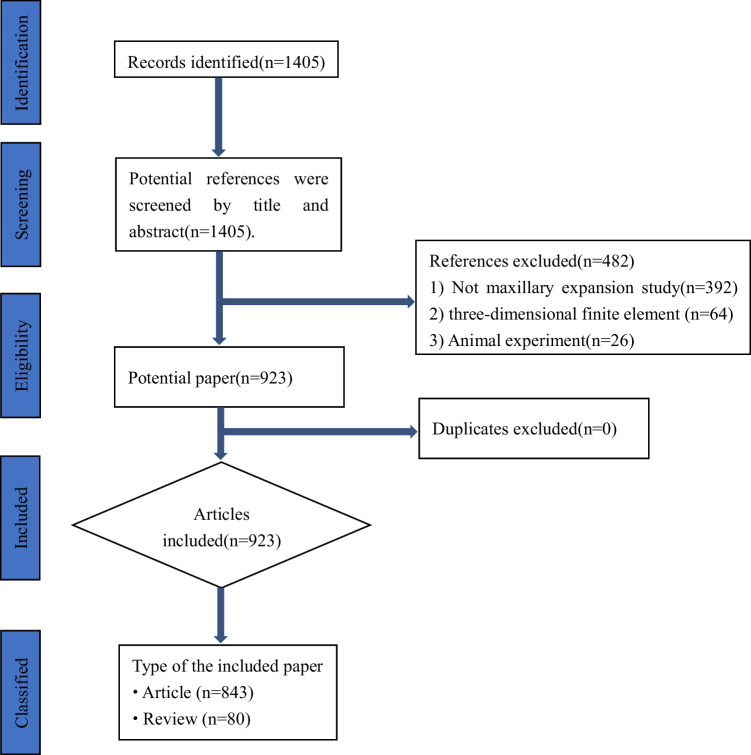
Flow diagram of the protocol of the study

Two independent investigators (Minmin Si and Hao Fan) excluded studies that did not meet the inclusion criteria by reviewing the titles and abstracts. The exclusion criteria were as follows: (1) studies that did not involve maxillary expansion or skeletal expansion, (2) simulated experiments such as three-dimensional finite element analysis, (3) animal experiments. In case of disagreements between the two independent investigators, a third researcher (Zhiyuan Feng) was consulted (Fig. 1).

#### Data import and processing

The final set of included literature was imported into CiteSpace 6.2.R4 software in plain text format for deduplication. The data processing involved a combination of software analysis and manual analysis. CiteSpace 6.2.R4 software was used for initial literature analysis, providing rankings and centrality measures for authors, countries, and institutions, as well as co-occurrence and clustering of keywords. To meet the output requirements (network size less than or equal to 300), this study gradually lowered the selection threshold after increasing the “Year Slice” from 1 to 2, until all criteria were met and the maximum visualization was achieved. The final software parameters were set as follows: “Time period = 1993–2023,” “Year Slice = 2,” “g-index = 10” (for author, country, institution, and keyword analysis), “Top N% = 5” (for reference and citing journal analysis). “Pathfinder” and “Pruning networks” were selected, while other parameters were set to default. VOS viewer software was used to optimize and enhance visualizations that were not aesthetically pleasing. Finally, relevant information was manually searched and supplemented in the table to achieve more comprehensive bibliometric results.

### Describing indicators

This study primarily presents the results in quantitative and percentage formats, as well as through visual network maps. The results include contribution and collaboration networks (authors, countries, and institutions), influence networks (references and journals), and keyword analysis (cluster and burst).

The visual network maps consist of nodes and links. Each node represents a project, and the size of the node corresponds to its frequency of occurrence. The links between nodes represent their collaborative relationships. Three different structural indicators are used to assess the quality of results: centrality, modularity, and silhouette. Centrality calculates the shortest paths between all pairs of nodes in the network to identify the influence of a node, particularly when it holds a central position within a cluster or serves as a bridge [9]. Nodes with high centrality are considered key points in the research [10].

Keyword clustering involves grouping strongly correlated nodes, revealing related research areas and their evolution over the years [11]. The modularity score (*Q* score) measures the quality of dividing the network into clusters. The *Q* score ranges from 0 to + 1, where a *Q* score > 0.3 indicates a significant clustering structure and a higher value indicates a well-structured network. Silhouette score (*S* score) evaluates the quality of the clustering configuration, ranging from − 1 to + 1. A network with an *S* score > 0.3, 0.5, or 0.7 is considered homogeneous, reasonable, or highly reliable, respectively [10].

## Results

A total of 923 articles were analyzed, consisting of 843 articles and 80 reviews.

### Analysis of annual publishing volume

**Fig. 2:**
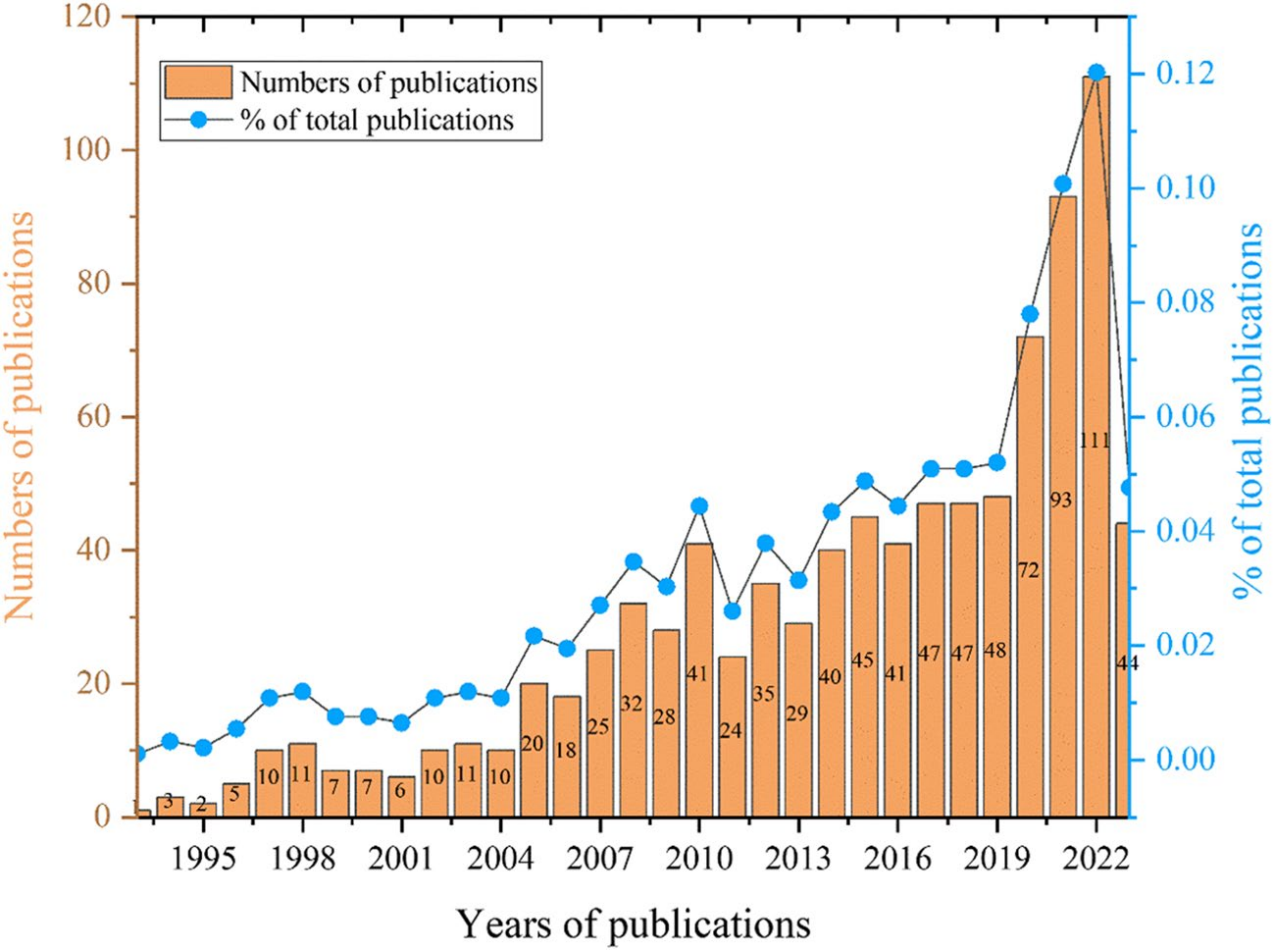
Annual changes in the number of publications

The annual publication output on maxillary skeletal expansion (Fig. 2) shows two distinct phases: (1) Before 2019, the annual publication volume followed an “S”-shaped trend; (2) after 2019, the annual publication volume increased exponentially, reaching its peak in 2022 with a count of 111 publications.

### Author, country, and institutional analysis

**Fig. 3:**
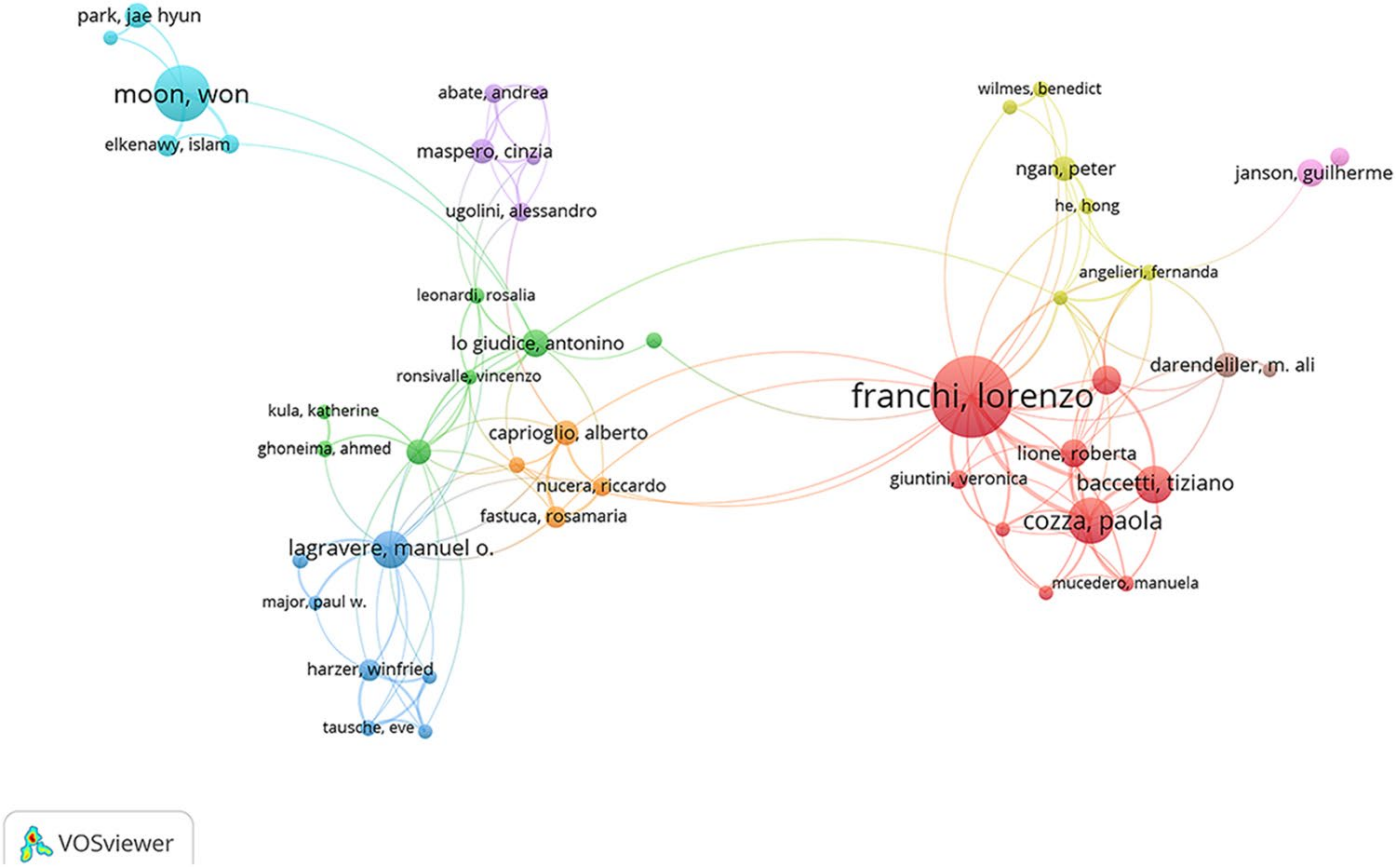
Main author co-occurrence network

The full names and abbreviations of the same authors were merged into a single author. Table 1 presents the top nine most productive authors, their publication counts, citation counts, and centrality scores, which are comprehensive data obtained from CiteSpace 6.2.R4 and VOS viewer. Among these nine authors, eight have centrality scores greater than 0, indicating a high level of scientific output consistent with extensive collaboration and communication. Figure 3 displays major author collaboration networks, with each color representing a network group where collaborations are more frequent. There is also some collaboration observed between different groups.

**Table 1:** Top nine most productive authors

Ranking	Name	Counts	Citations	Centrality	Country
1	Franchi, Lorenzo	43	1914	0.08	Italy
2	Baccetti, Tiziano	25	1703	0.01	Italy
3	Moon, Won	20	528	0.01	USA
4	Cozza, Paola	17	628	0.01	Italy
5	Lagravere, Manuel O	17	489	0.04	Canada
6	Mcnamara, JA	17	1570	0.01	USA
7	Lo Giudice, Antonino	8	119	0.03	Italy
8	Elkenawy, Islam	8	140	0.01	USA
9	Ngan, Peter	8	565	0	USA

*USA*, United States of America

**Fig. 4:**
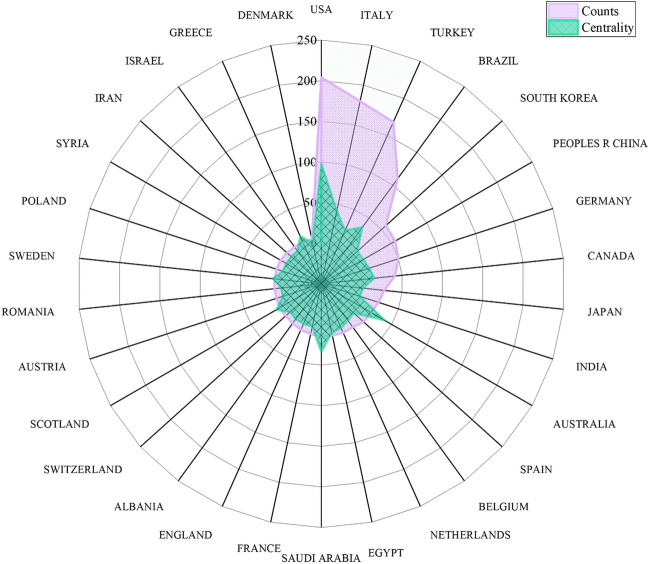
The 30 countries with publication volumes greater than 5 and their centrality scores. Due to the significant difference in values between publication quantity and centrality scores, the centrality scores are multiplied by 200 to achieve a more prominent visual effect

**Fig. 5:**
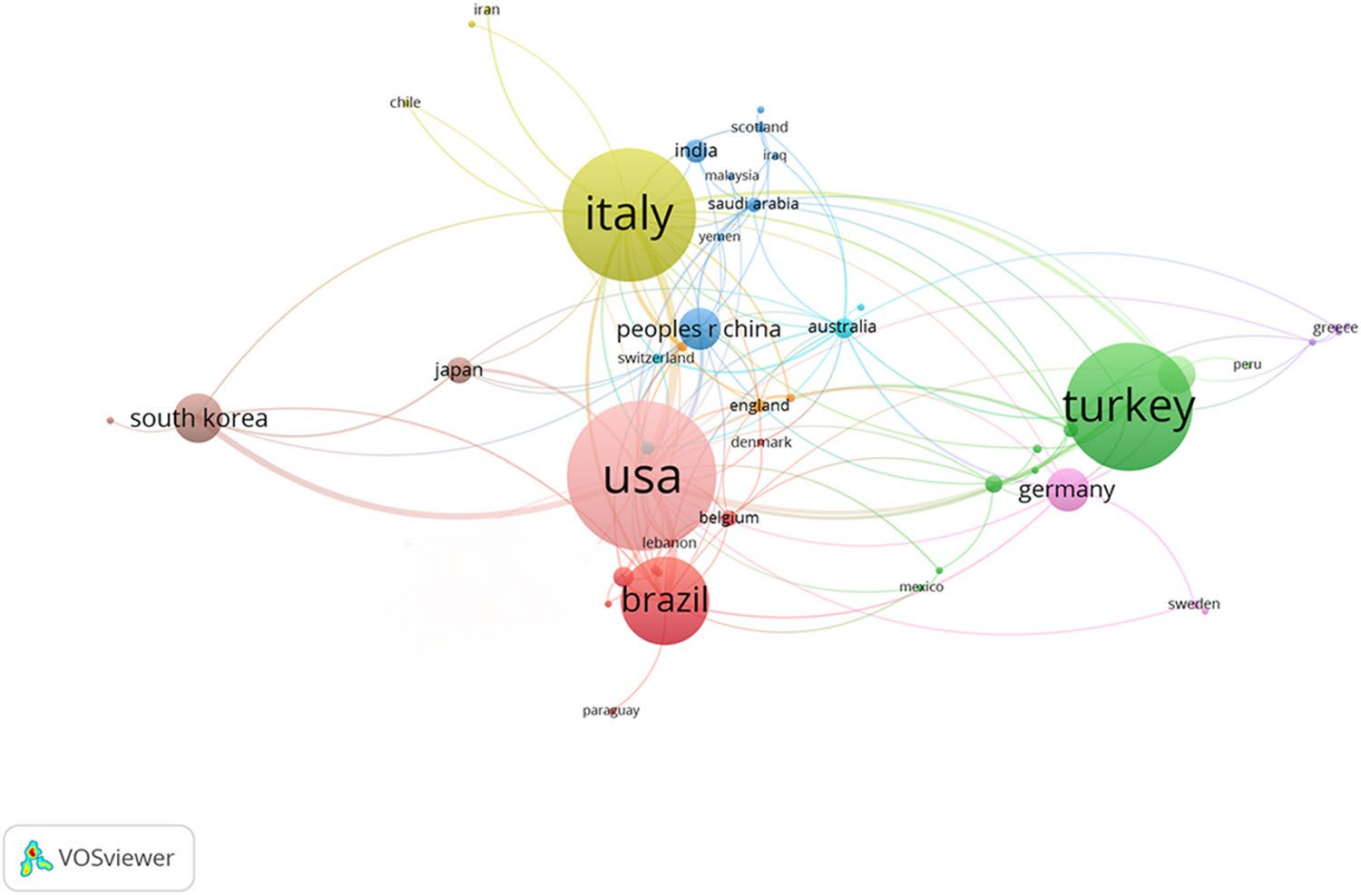
Main country co-occurrence network

VOS viewer analysis revealed the involvement of 63 countries in research production related to maxillary skeletal expansion. Figure 4 illustrates the top 30 countries in terms of publication count and their centrality scores. The USA ranks first with over 200 publications (22% of the total), followed by Italy, Turkey, Brazil, South Korea, and others. There is close collaboration among different countries, providing a solid foundation for research and development in the field of maxillary skeletal expansion (Fig. 5).

**Fig. 6:**
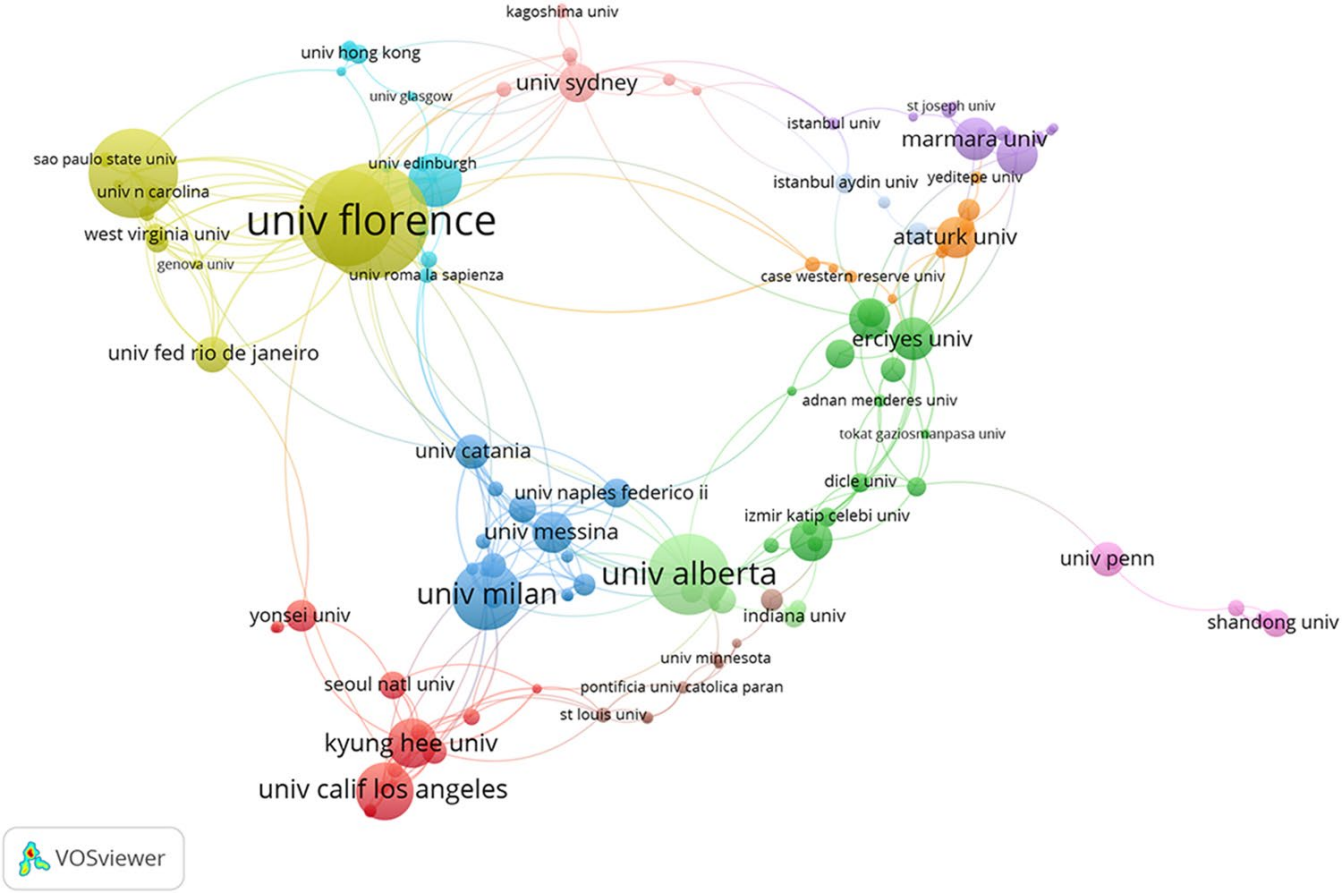
Main institution co-occurrence network

**Fig. 7:**
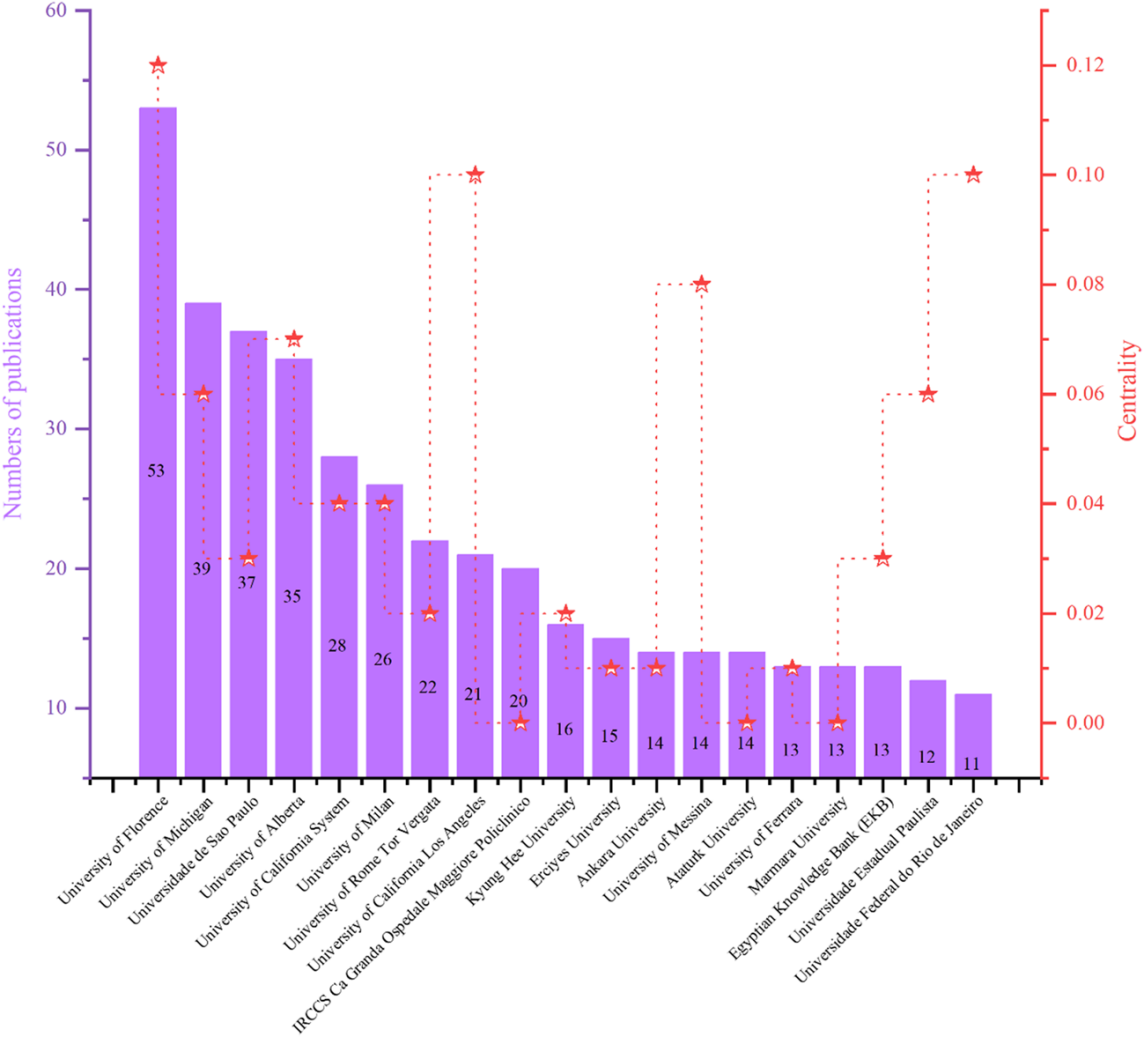
Nineteen institutions with publication volumes greater than 10

From an institutional perspective, a total of 809 institutions have been involved in research in this field. Figure 6 displays 160 research institutions that have appeared in the literature at least three times. Among the top ten institutions in terms of publication count, four are from the USA, and three are from Italy. The University of Florence ranks first in both publication count and centrality score (Fig. 7). This indicates the significant contributions made by the USA and Italy in the research on maxillary skeletal expansion, which aligns with the analysis of the countries mentioned earlier. Figure 6 shows major collaboration networks, with most collaborations occurring among institutions within the same country.

### Keyword analysis

#### Keyword co-occurrence

**Fig. 8:**
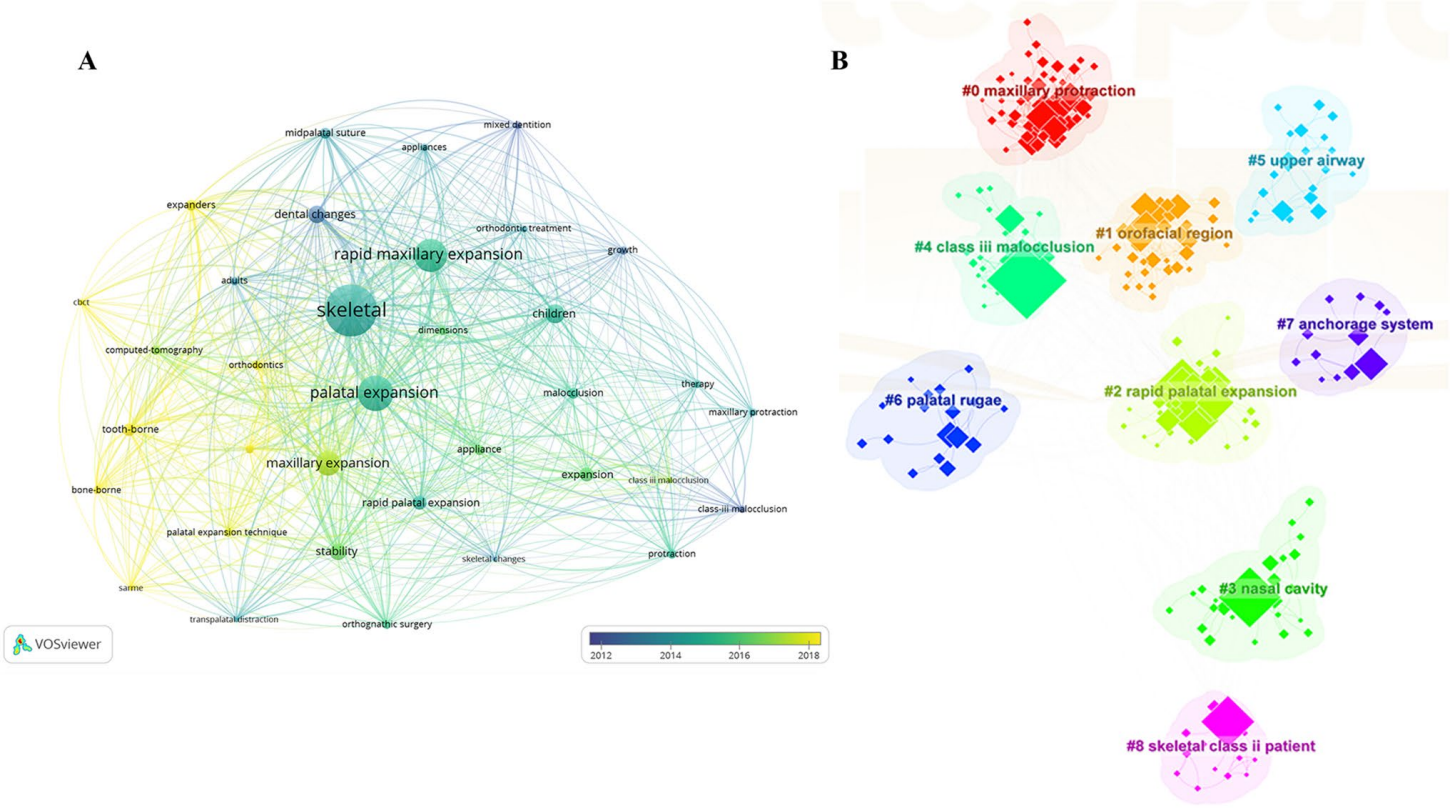
**A** The co-occurrence of keywords that appear more than 30 times. **B** Cluster graph of keywords

Keywords represent the hotspots and trends in a research field [8]. A total of 1862 keywords were identified in the included literature, and Fig. 8A displays keywords that appear more than 30 times. The top three most frequently occurring keywords are “skeletal,” “palatal expansion,” and “rapid maxillary expansion.” The top three keywords in terms of centrality are “children,” “class III malocclusion,” and “skeletal,” which align with the theme of this study. When observing the keywords from right to left, the changes over the years include “growth,” “mixed dentition,” “class III occlusion,” “rapid maxillary expansion,” “adult,” “Orthognathic surgery,” “tooth-borne,” and “bone-borne.” Combining this analysis with other data, we can conclude that research on the indications for maxillary expansion has gradually expanded from children in the growth period to adults. Maxillary expansion is frequently applied in Class III malocclusions. Comparative studies on the effects of tooth-borne and bone-borne maxillary expansion have increased since 2018.

### Keywords with citation burst

**Fig. 9:**
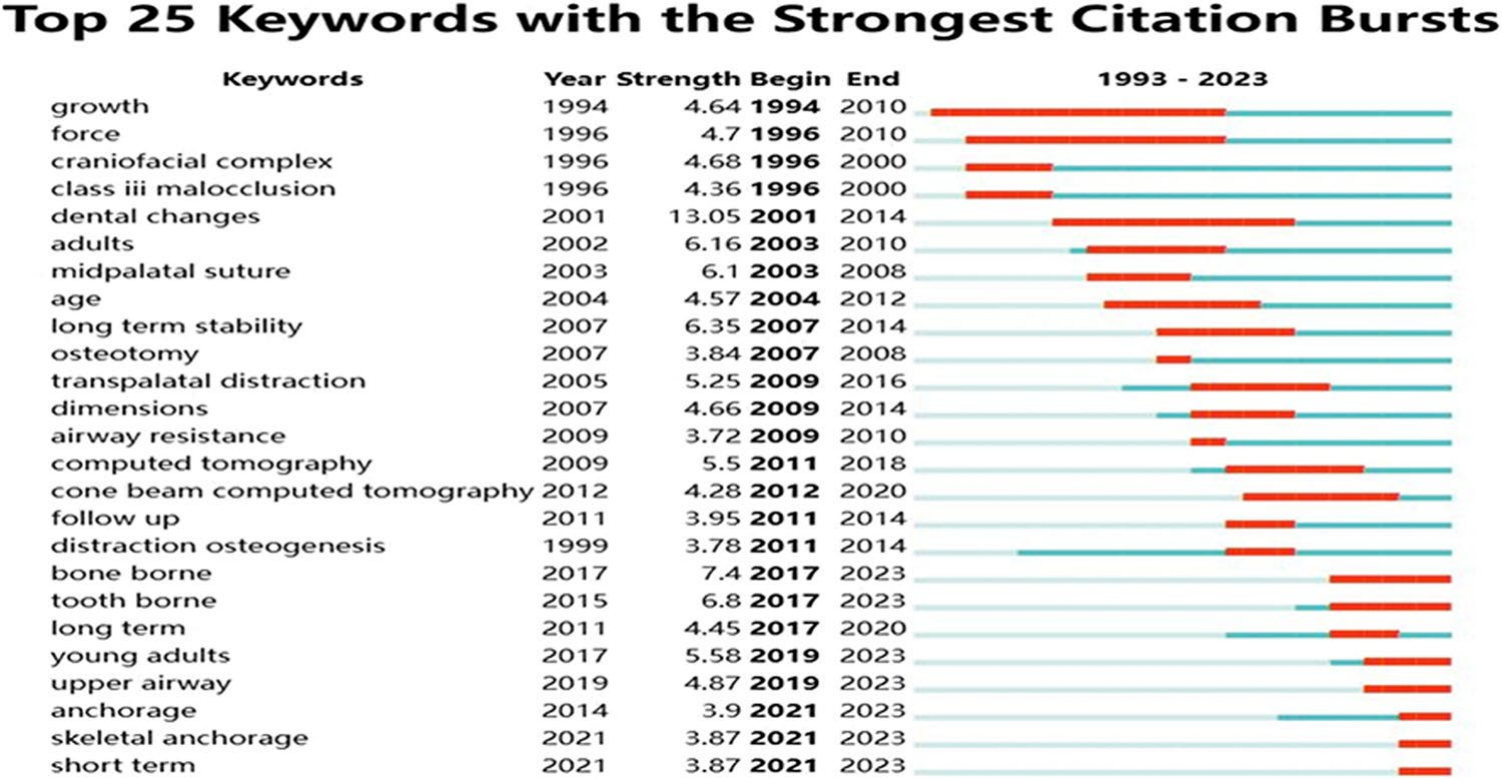
Top 25 keywords with strongest citation bursts, the red line indicating years of frequent appearances and the green line indicating years with fewer appearances

Keywords with citation bursts indicate that specific keywords have a high citation rate during certain periods, revealing the research hotspots in a particular research field during specific times [12]. These newly emerging keywords can be identified through burst maps. The year when keywords began to receive high citations (Fig. 9) can be divided into two phases: (1) Before 2010, there was a significant amount of research on the appropriate age for maxillary expansion, the relationship between the palatal suture and growth, and the impact of maxillary expansion on airway resistance, which started to attract widespread attention from scholars. (2) After 2010, with advancements in technology, cone beam computed tomography (CBCT) has been increasingly applied in orthodontic clinical research. Scholars have extensively explored the effects of various forms of anchorage (primarily tooth-borne and bone-borne) in both short-term and long-term applications of maxillary expansion, gradually extending their application to young adults. Simultaneously, research on the impact on the airway continues to deepen.

### Keyword clustering

We extracted cluster labels from keywords related to the article titles, resulting in a total of nine distinct clusters. The clustering results are displayed in Fig. 8B. Some of the cluster labels include “maxillary protraction,” “orofacial region,” “rapid palatal expansion,” and “nasal cavity,” among others.

### Cited journal analysis

Table 2 presents the top ten journals ranked by citation count, primarily distributed in the USA, the UK, and Germany, with the USA having four journals in the list. The *American Journal of Orthodontics and Dentofacial Orthopedics*, *Angle Orthodontist*, and *European Journal of Orthodontics* rank in the top three positions and are considered authoritative journals in the field of orthodontics. The average impact factor of these journals ranges from 2 to 3, suggesting that researchers may need to further explore and conduct research in this field.

**Table 2:** The top ten journals related to maxillary skeletal expansion

Ranking	Journal	Counts	Impact Factor	H-Index	Country	Centrality
1	American Journal of Orthodontics and Dentofacial Orthopedics	1281	3.0	105	USA	0.7
2	Angle Orthodontist	850	3.4	75	USA	0.46
3	European Journal of Orthodontics	681	2.6	75	England	0.18
4	Journal of Oral and Maxillofacial Surgery	291	1.9	109	USA	0.37
5	Journal of Clinical Orthodontics	261	0	–-	USA	0.02
6	Progress in Orthodontics	254	4.8	22	Germany	0.02
7	International Journal of Oral and Maxillofacial Surgery	242	2.4	90	England	0.2
8	Seminars in Orthodontics	222	4.2	39	USA	0
9	Journal of Orofacial Orthopedics-Fortschritte der Kieferorthopadie	205	1.7	39	Germany	0.1
10	Journal of Cranio-Maxillofacial Surgery	191	3.1	67	USA	0.01

### Reference analysis

Table 3 presents the top ten cited references, all of which were published between 2015 and 2018. Among them, nine are studies on maxillary expansion assisted by mini-implant anchorage. It can be inferred that mini-implant anchorage-assisted maxillary expansion sparked great research interest among scholars during the period of 2015–2018 and served as a reference for subsequent studies. This is one of the reasons for the exponential increase in annual publications after 2019 (Fig. 2). Moreover, the authors of these ten references come from the USA, South Korea, and Brazil, all of which are countries with a high publication output. This further demonstrates the significant contributions of these countries in exploring new research areas, strengthening international collaboration, and promoting the development of maxillary expansion.

**Table 3:** The top ten cited articles related to maxillary skeletal expansion

Ranking/title	Year	Author/country	citations	Journal	Centrality
1. Skeletal and dentoalveolar changes after cc in young adults: A cone-beam computed tomography study	2017	Jung Jin Park (Korea); Ji Hyun Tahk (USA)	50	Korean Journal of Orthodontics	0.09
2. Changes in the midpalatal and pterygopalatine sutures induced by micro-implant-supported skeletal expander, analyzed with a novel 3D method based on CBCT imaging	2017	Daniele Cantarella (USA)	49	Progress in Orthodontics	0.04
3. Non-surgical treatment of transverse deficiency in adults using Microimplant-assisted Rapid Palatal Expansion (MARPE)	2017	Daniel Paludo Brunetto (Brazil); Won Moon (USA)	42	Dental Press Journal of Orthodontics	0.05
4. Microimplant-assisted rapid palatal expansion appliance to orthopedically correct transverse maxillary deficiency in an adult	2016	Chuck Carlson (USA); Andre Wilson Machado (Brazil)	41	American Journal of Orthodontics and Dentofacial Orthopedics	0.33
5. Effects of monocortical and bicortical mini-implant anchorage on bone-borne palatal expansion using finite element analysis	2017	Robert J. Lee (USA)	33	American Journal of Orthodontics and Dentofacial Orthopedics	0.04
6. Evaluation of miniscrew-supported rapid maxillary expansion in adolescents: A prospective randomized clinical trial	2018	Tugce Celenk-Koca (Netherlands); Aslihan Ertan Erdinc (Turkey); Serpil Hazar (USA)	33	American Journal of Orthodontics and Dentofacial Orthopedics	0.12
7. Tooth-borne vs bone-borne rapid maxillary expanders in late adolescence	2015	Lu Lin (Korea)	32	Angle Orthodontist	0.11
8. Nonsurgical miniscrew-assisted rapid maxillary expansion results in acceptable stability in young adults	2016	Sung-Hwan Choi (Korea)	32	Angle Orthodontist	0.09
9. Stability of dental, alveolar, and skeletal changes after miniscrew-assisted rapid palatal expansion	2017	Hyun-Mook Lim (Korea)	30	Korean Journal of Orthodontics	0
10. Midfacial changes in the coronal plane induced by microimplant-supported skeletal expander, studied with cone-beam computed tomography images	2018	Daniele Cantarella (USA)	28	American Journal of Orthodontics and Dentofacial Orthopedics	0.19

Only one author per country is shown in the table

In 2015, Korean scholars compared the effects of tooth-borne and bone-borne maxillary expansion on dental and skeletal changes in late adolescence. The results showed that bone-borne expanders produced greater skeletal effects and fewer dental side effects [1]. This finding stimulated scholars to consider and explore skeletal expansion in the late growth period and even in adulthood.

## Discussion

The Web of Science Core Collection was chosen as the main database for retrieval because it is one of the preferred databases for scientific bibliometric analysis, providing comprehensive coverage of references and citations [11]. Regarding the search strategy, the aim of this study was to analyze research on maxillary skeletal expansion, and “maxillary,” “skeletal,” and “expansion” are essential components of the search query, with each term being necessary. Therefore, the search terms used were “TS = maxillary expansion” and “TS = skeletal expansion,” linked by the Boolean operation symbol “AND” to improve precision.

The number of publications within a specific time period reflects the research development trends and pace of a discipline [13]. The increasing number of publications on maxillary skeletal expansion, with a nearly doubling of publications every decade, follows the classic description of scientific temporal development [14]. Additionally, the significant increase in publication volume after 2019 may be highly correlated with the broader indications and widespread research interest in skeletal expansion following the emergence of skeletal anchorage techniques.

Lorenzo Franchi, Tiziano Baccetti, and Paola Cozza, who are from Italy, have been conducting research in the field since 2002, and their research interests align with the developmental trends of maxillary skeletal expansion. In a previous bibliometric analysis conducted by our research group on maxillary protraction, Lorenzo Franchi and Tiziano Baccetti ranked first and third in terms of publication volume [15]. This indicates the close association between maxillary protraction and expansion and highlights the key roles these two authors have in both research areas. Further analysis of their research content reveals that Italian scholars primarily explore the effectiveness of maxillary expansion with or without anterior protraction in patients during the growth period or mixed dentition phase. They have produced numerous clinical trial studies and review papers [16–18], establishing a solid theoretical foundation for the further development of maxillary expansion. Won Moon, from the USA, started his research relatively later (in 2014), but has a high publication volume, focusing specifically on the effects of micro-implant-assisted rapid palatal expansion (MARPE) in non-growing patients. Looking back at his research trajectory, Won Moon’s team has conducted extensive studies on MARPE, ranging from initial three-dimensional finite element models [19] and case reports on MARPE application in adult patients [20] to more mature clinical controlled trials [21]. Their research has affirmed the benefits of MARPE in improving respiration and made significant contributions to the rapid development of maxillary skeletal expansion. It is worth noting that the exclusion of simulations such as three-dimensional finite element models in this study may have somewhat weakened the research achievements of Won Moon. In summary, the collaboration and complementarity between authors from Italy and the USA have contributed to the steady advancement of this field.

High centrality keywords such as “children,” “class III malocclusion,” and “skeletal” indicate that these keywords play a bridging role in the field. The *Q* value of keyword clustering is 0.4464 > 0.3, and the *S* value is 0.7266 > 0.5, indicating that the clustering effect is significant and the data is convincing [15].

Based on the clustering results and incorporating other keyword information, the discussion can focus on the following aspects:

### #0 maxillary protraction and #1 orofacial region—combining maxillary expansion with anterior traction improves skeletal and facial structures

The reappearance of the label “maxillary protraction” confirms that there is significant research on the combined use of maxillary protraction and expansion. Further analysis of the keywords within each cluster reveals common terms such as “class III malocclusion,” “face mask therapy,” “expansion,” and “orthopedic treatment.” Among class III malocclusion, maxillary deficiency in three-dimensional development is often present. Therefore, in clinical practice, maxillary expansion combined with protraction is frequently employed to achieve orthopedic treatment. The research primarily focuses on the effectiveness of maxillary skeletal expansion in facilitating protraction [22, 23]. This aligns with the findings of the previous bibliometric analysis conducted by our research group, which indicated that the use of maxillary expansion should be based on clinical indications rather than solely promoting maxillary advancement [15].

### #2 rapid palatal expansion and #7 anchorage system—bone-borne RPE is suitable for adults

The purpose of maxillary expansion is to achieve maximum dental arch alignment and minimize tooth movement. The most widely used treatment approach for maxillary expansion in orthodontic therapy is rapid maxillary expansion (RME) performed in early adolescence, typically between the ages of 10 and 13 [24, 25]. Both rapid and slow expansion techniques can yield certain effects, with RME being relatively more effective in increasing the posterior width of the maxilla, while slow maxillary expansion (SME) results in minimal tipping of the molars [26]. The impact of different types of anchorage on the palate and teeth is particularly noteworthy [27]. The keyword “borne” in Cluster 7 suggests the types of anchorage used (Fig. 8B). Traditional tooth-borne expanders rely on posterior teeth for anchorage, which can lead to tilting of the teeth and alveolar bone along with the opening of the midpalatal suture [24, 28]. Tooth-bone mixed-borne (TBB) design involves bonding the RME appliance to the posterior teeth and utilizing two mini-implants anchored in the palate. For pediatric patients with unfused midpalatal suture, the choice of different types of anchorage, such as tooth-borne or bone-borne, does not significantly impact the expansion outcomes [28]. However, local anesthesia is required for the insertion and removal of mini-implants in the TBB group, which may be an unpleasant experience for younger patients before adolescence. Additionally, the expansion outcomes with mixed-borne design are similar to those achieved with traditional tooth-borne approaches, making bone-borne methods not recommended for early adolescence applications.

As patients reach more advanced stages of growth and development, conventional palatal expanders become increasingly challenging to separate the midpalatal suture [25]. In recent years, orthodontists have been incorporating bone-borne palatal expanders into their treatment approaches [25]. Micro-implant-assisted rapid palatal expansion (MARPE) has been utilized in late adolescence and adults with transverse maxillary deficiency [29]. MARPE, designed with two or four mini-implants, applies the expansion force directly to the maxilla and midpalatal suture, allowing for more orthopedic changes and reducing the side effects on teeth and alveolar bone [30]. While the degree of ossification of the midpalatal suture and other cranial sutures increases with age, the increased bone density contributes to the stability of mini-implant insertion. Compared to traditional rapid palatal expansion (RPE) performed in the early mixed dentition stage [31], MARPE provides opportunities for midpalatal suture opening, extending the indications for maxillary expansion to late adolescence and even some adults. The success rate of miniplate-supported rapid palatal expansion (MARPE) devices in late adolescence and young adults for maxillary skeletal expansion is significantly high, with increased midpalatal suture opening, fewer dental side effects, and more pronounced orthopedic responses, thereby defining the scope of maxillary skeletal expansion. A systematic review reported that bone-borne rapid expansion methods produced greater skeletal effects in late adolescence expansion treatment compared to traditional tooth-borne expanders [32]. When comparing the use of four mini-implant-supported bone-borne MARPE with traditional tooth-borne expanders, studies have found that MARPE increases the extent of skeletal changes by 1.5 to 2.8 times that of tooth-borne expansion [33].

For adult patients with mature skeletal structures and cranial sutures, surgical-assisted rapid maxillary expansion (SARME) can be performed to reduce the risks associated with orthognathic surgery alone. SARME is typically recommended for correcting maxillary transverse deficiencies greater than 5 mm, as it overcomes the resistance of the midpalatal suture, thus increasing the potential for expansion [34, 35]. In SARME treatment, the rapid expansion effects achieved with tooth-borne or bone-borne designs are similar after surgically assisted midpalatal suture opening [36]. Compared to MARPE, SARME is invasive, expensive, associated with surgical complications, and may lead to undesired issues such as asymmetrical expansion, incisor discoloration, and periodontal complications during maxillary expansion [35]. Therefore, the significant increase in the number of articles and their proportion on MARPE published after 2019 suggests that MARPE is a promising non-surgical method for addressing maxillary transverse deficiencies in adults and achieving skeletal expansion. However, the effectiveness of MARPE varies among different patients, and its success rate is influenced by various factors such as the degree of midpalatal suture fusion, circummaxillary suture widths, palatal bone thickness, and implant positioning [37]. Hence, it is necessary to evaluate the potential influencing factors of MARPE from multiple perspectives, and further research is needed to investigate the impact of different factors on the success rate of MARPE.

### #3nasal cavity and #5 upper airway—maxillary expansion increases nasal cavity and upper airway volume, improving breathing

The keywords “pharyngeal airway,” “acoustic rhinometry,” “volume changes,” “obstructive sleep apnea,” and “improvement” in two clusters suggest that maxillary skeletal expansion has an impact on nasal cavity and pharyngeal airway volume and may potentially improve respiratory disorders such as obstructive sleep apnea (OSA). Acoustic rhinometry is a reliable and objective method used to measure the relationship between the nasal cavity cross-sectional area and the distance of entry into the nasal cavity, assessing the geometric shape of the nose [38, 39]. This may explain the emphasis on nasal studies. Based on this concept, this study further screened relevant literature and conducted analysis.

Early studies indicated that after rapid palatal expansion (RPE) in growing children, nasal width [40], volume [41], and minimum cross-sectional area increased [38], and nasal airway resistance decreased [42], thereby improving nasal breathing [39, 43]. With more studies on adult expansion and its effects, similar improvements in nasal parameters were observed with maxillary skeletal expansion techniques such as SARME and MARPE [44, 45], extending to the entire upper airway (including nasopharynx and oropharynx) and the nasal and facial regions. Most studies indicate that maxillary skeletal expansion has no significant impact on the oropharynx but can increase nasopharyngeal airway volume [46–48]. Additionally, there is ongoing debate regarding whether bone-borne maxillary expansion can reduce nasal septum deviation. Several studies from a decade ago suggested no effect on the position of the nasal septum [49, 50], while recent studies indicate a reduction in nasal septum deviation, leading to improved facial aesthetics [45, 51, 52]. Further research is needed to determine the true effectiveness of maxillary skeletal expansion in reducing nasal septum deviation and its impact on facial aesthetics and function.

Furthermore, the association between maxillary constriction and obstructive sleep apnea (OSA) has drawn attention in this study, as maxillary constriction may be an etiological factor [53]. A recent study demonstrated that maxillary expansion significantly increased airway volume and nasal width, with the latter showing a significant correlation with improvements in OSA-related measures [54]. In the future, maxillary expansion appears to be a potential approach for treating OSA. It is important to note that when using maxillary expansion solely for respiratory disorders, there should be orthodontic indications such as maxillary transverse narrowness [55].

### #4 class III malocclusion and #8 skeletal class II patient—maxillary skeletal expansion improves function and aesthetics

In conclusion, research on maxillary skeletal expansion for the treatment of maxillary transverse deficiency has shown a growing trend over the years. Its effectiveness has been demonstrated not only in treating Class III patients but also in Class II patients. Patients with maxillary constriction have experienced functional and aesthetic improvements following skeletal expansion. In terms of function, potential changes may include (1) increased intercanine and intermolar width of the mandible[56], (2) increased maxillary arch width and uprighting of mandibular posterior teeth [57], (3) restoration of abnormal condylar position of the mandible [58], (4) the opportunity to improve Class II molar occlusion [59], (5) establishment of normal and stable oro-facial function [58], (6) elevation of the tongue position [60], and proper swallowing space [61]. In terms of aesthetics, maxillary skeletal expansion contributes to the harmonious width of the midface and subnasal soft tissues.

## Conclusion

1. MARPE (miniscrew-assisted rapid palatal expansion) and SARME (surgically assisted rapid maxillary expansion) have gained widespread attention and become research hotspots due to their applicability in adults whose growth and development have ceased, while still producing favorable skeletal effects.
2. In addition to widening the maxillary arch, maxillary expansion techniques have shown significant effects on increasing nasal cavity width and volume. However, there is still controversy regarding whether they can effectively improve the deviated nasal septum.
3. Maxillary skeletal expansion techniques have been shown to increase upper airway volume and improve breathing, making them potentially valuable in the treatment of obstructive sleep apnea (OSA).

## Advantages and limitations

This study has several strengths. Firstly, it utilizes two novel analytical methods to provide insights into the evolving research trends over time, visualizes author, country, and institution networks, and goes beyond common metrics used in bibliometric analysis, such as impact factor, H-index, and citation counts. Secondly, the study combines automated software analysis with a manual examination of the literature, ensuring comprehensive and accurate analysis. However, the study exclusively relies on Web of Science Core Collection (WoSSC) as the primary data source, as many existing biomedical and life science databases like PubMed or Embase do not provide full-text and citation analysis. This may to some extent result in insufficient data sources.

## Data Availability

Data will be available upon reasonable request.
